# Supplementary material for: HIVprotI: an integrated web based platform for prediction and design of HIV proteins inhibitors
Source: J Cheminform. 2018 Mar 9;10:12. doi: 10.1186/s13321-018-0266-y (PMC5845081; doi:10.1186/s13321-018-0266-y)
Supplement: Supplementary file 2 — Additional file 2. Source code of HIVProtI web server. [file 13321_2018_266_MOESM2_ESM.zip › HIVprotI_Source-code/hivprotI/weka-3-6-6/doc/weka/associations/tertius/Literal.html]

Literal


---


|  |  |  |
| --- | --- | --- |
| **Overview** \| **Package** \| **Class** \| **Tree** \| **Deprecated** \| **Index** \| **Help** \| **Weka's home** | |  |
| **PREV CLASS**   **NEXT CLASS** | **FRAMES**    **NO FRAMES**     **All Classes** |  |
| SUMMARY: NESTED \| FIELD \| CONSTR \| METHOD | DETAIL: FIELD \| CONSTR \| METHOD |  |


---


## weka.associations.tertius Class Literal

```
java.lang.Object
  weka.associations.tertius.Literal
```

**All Implemented Interfaces:**: java.io.Serializable, RevisionHandler

**Direct Known Subclasses:**: AttributeValueLiteral

---

``` public abstract class Literal extends java.lang.Object implements java.io.Serializable, RevisionHandler ```

**Version:**
:   $Revision: 1.5 $

**Author:**
:   Peter A. Flach, Nicolas Lachiche

**See Also:**: Serialized Form

---

| **Field Summary** | |
| --- | --- |
| `static int` | `NEG` |
| `static int` | `POS` |


**Constructor Summary**

`Literal(Predicate predicate,
int sign,
int missing)`


| **Method Summary** | |
| --- | --- |
| `Literal` | `getNegation()` |
| `Predicate` | `getPredicate()` |
| `abstract  boolean` | `negationSatisfies(Instance instance)` |
| `boolean` | `negative()` |
| `boolean` | `positive()` |
| `abstract  boolean` | `satisfies(Instance instance)` |
| `void` | `setNegation(Literal negation)` |
| `abstract  java.lang.String` | `toString()` |

**Methods inherited from class java.lang.Object**

`equals, getClass, hashCode, notify, notifyAll, wait, wait, wait`

**Methods inherited from interface weka.core.RevisionHandler**

`getRevision`

**Field Detail**

### NEG

```
public static final int NEG
```

**See Also:**: Constant Field Values

---


### POS

```
public static final int POS
```

**See Also:**: Constant Field Values


**Constructor Detail**

### Literal

```
public Literal(Predicate predicate,
               int sign,
               int missing)
```


**Method Detail**

### getPredicate

```
public Predicate getPredicate()
```

---


### getNegation

```
public Literal getNegation()
```

---


### setNegation

```
public void setNegation(Literal negation)
```

---


### positive

```
public boolean positive()
```

---


### negative

```
public boolean negative()
```

---


### satisfies

```
public abstract boolean satisfies(Instance instance)
```

---


### negationSatisfies

```
public abstract boolean negationSatisfies(Instance instance)
```

---


### toString

```
public abstract java.lang.String toString()
```

:   **Overrides:**: `toString` in class `java.lang.Object`


---


|  |  |  |
| --- | --- | --- |
| **Overview** \| **Package** \| **Class** \| **Tree** \| **Deprecated** \| **Index** \| **Help** \| **Weka's home** | |  |
| **PREV CLASS**   **NEXT CLASS** | **FRAMES**    **NO FRAMES**     **All Classes** |  |
| SUMMARY: NESTED \| FIELD \| CONSTR \| METHOD | DETAIL: FIELD \| CONSTR \| METHOD |  |


---
